# Molecular evolutionary analysis of novel NSP4 mono-reassortant G1P[8]-E2 rotavirus strains that caused a discontinuous epidemic in Japan in 2015 and 2018

**DOI:** 10.3389/fmicb.2024.1430557

**Published:** 2024-07-10

**Authors:** Yoshiki Fujii, Takeshi Tsugawa, Yuya Fukuda, Shuhei Adachi, Saho Honjo, Yusuke Akane, Kenji Kondo, Yoshiyuki Sakai, Toju Tanaka, Toshiya Sato, Yoshihito Higasidate, Noriaki Kubo, Toshihiko Mori, Shinsuke Kato, Ryo Hamada, Masayoshi Kikuchi, Yasuo Tahara, Kazushige Nagai, Toshio Ohara, Masaki Yoshida, Shuji Nakata, Atsuko Noguchi, Wakako Kikuchi, Hiromichi Hamada, Shoko Tokutake-Hirose, Makoto Fujimori, Masamichi Muramatsu

**Affiliations:** ^1^Department of Virology II, National Institute of Infectious Diseases, Tokyo, Japan; ^2^Department of Pediatrics, Sapporo Medical University School of Medicine, Hokkaido, Japan; ^3^Department of Pediatrics, Hakodate Municipal Hospital, Hokkaido, Japan; ^4^Department of Pediatrics, National Hospital Organization Hokkaido Medical Center, Hokkaido, Japan; ^5^Department of Pediatrics, Iwamizawa Municipal General Hospital, Hokkaido, Japan; ^6^Department of Pediatrics, Japan Community Health Care Organization Sapporo Hokushin Hospital, Hokkaido, Japan; ^7^Department of Pediatrics, Japan Red Cross Urakawa Hospital, Hokkaido, Japan; ^8^Department of Pediatrics, NTT Medical Center Sapporo, Hokkaido, Japan; ^9^Department of Pediatrics, Rumoi City Hospital, Hokkaido, Japan; ^10^Department of Pediatrics, Sunagawa City Medical Center, Hokkaido, Japan; ^11^Department of Pediatrics, Steel Memorial Muroran Hospital, Hokkaido, Japan; ^12^Department of Pediatrics, Takikawa Municipal Hospital, Hokkaido, Japan; ^13^Department of Pediatrics, Tomakomai City Hospital, Hokkaido, Japan; ^14^Department of Pediatrics, Yakumo General Hospital, Hokkaido, Japan; ^15^Nakata Pediatric Clinic, Hokkaido, Japan; ^16^Department of Pediatrics, Akita University Graduate School of Medicine, Akita, Japan; ^17^Department of Pediatrics, Tokyo Women's Medical University Yachiyo Medical Center, Chiba, Japan; ^18^Department of Infectious Disease Research, Institute of Biomedical Research and Innovation, Foundation for Biomedical Research and Innovation at Kobe, Hyogo, Japan

**Keywords:** rotavirus, molecular epidemiology, next-generation sequencing, Japan, reassortment, evolution

## Abstract

In the 2010s, several unusual rotavirus strains emerged, causing epidemics worldwide. This study reports a comprehensive molecular epidemiological study of rotaviruses in Japan based on full-genome analysis. From 2014 to 2019, a total of 489 rotavirus-positive stool specimens were identified, and the associated viral genomes were analyzed by next-generation sequencing. The genotype constellations of those strains were classified into nine patterns (G1P[8] (Wa), G1P[8]-E2, G1P[8] (DS-1), G2P[4] (DS-1), G3P[8] (Wa), G3P[8] (DS-1), G8P[8] (DS-1), G9P[8] (Wa), and G9P[8]-E2). The major prevalent genotype differed by year, comprising G8P[8] (DS-1) (37% of that year’s isolates) in 2014, G1P[8] (DS-1) (65%) in 2015, G9P[8] (Wa) (72%) in 2016, G3P[8] (DS-1) (66%) in 2017, G1P[8]-E2 (53%) in 2018, and G9P[8] (Wa) (26%) in 2019. The G1P[8]-E2 strains (G1-P[8]-I1-R1-C1-M1-A1-N1-T1-E2-H1) isolated from a total of 42 specimens in discontinuous years (2015 and 2018), which were the newly-emerged NSP4 mono-reassortant strains. Based on the results of the Bayesian evolutionary analyses, G1P[8]-E2 and G9P[8]-E2 were hypothesized to have been generated from distinct independent inter-genogroup reassortment events. The G1 strains detected in this study were classified into multiple clusters, depending on the year of detection. A comparison of the predicted amino acid sequences of the VP7 epitopes revealed that the G1 strains detected in different years encoded VP7 epitopes harboring distinct mutations. These mutations may be responsible for immune escape and annual changes in the prevalent strains.

## 1 Introduction

Rotavirus A (RVA) is the major cause of gastroenteritis in infants and young children worldwide. In 2017–2018, RVA caused an estimated approximately 200,000 deaths globally in children under five years of age (Cohen et al., 2022). Although most RVA-related deaths occur in developing countries, RVA also imposes a substantial burden in developed countries, including Japan (Tate et al., 2016; Burnett et al., 2020; Tsugawa et al., 2021; Hallowell et al., 2022). Two live attenuated RVA vaccines were introduced in Japan in 2011 (Rotarix^®^, GlaxoSmithKline Biologicals, Rixensart, Belgium) and in 2012 (RotaTeq^®^, Merck & Co., Inc., Kenilworth, NJ) and included in the routine vaccination program since October 2020. RVA vaccines are effective; however, selective pressure on a vaccine may induce an epidemic strain shift. To monitor Japanese RVA epidemic strains, since 2012, our laboratory has conducted RVA surveillance based on full-genome analysis (Fujii et al., 2014, 2019, 2020).

The RVA genome consists of 11 double-stranded RNA segments that encode six structural proteins (VPs) and six nonstructural proteins (NSPs) (Estes and Greenberg, 2013). RVAs are assigned to specific genotypes based on the classification of each of the 11 genome segments, which is determined by sequence conservation, with distinctions established according to predefined nucleotide sequence identity cutoff values (Matthijnssens et al., 2008a,b; Matthijnssens et al., 2011). The VP7-VP4-VP6-VP1-VP2-VP3-NSP1-NSP2-NSP3-NSP4-NSP5/6 -encoding genes of the RVA strains are designated using the abbreviations Gx-P[x]-Ix-Rx-Cx-Mx-Ax-Nx-Tx-Ex-Hx (where x = genotype number), respectively. Human RVAs are primarily classified into three genogroups based on their genotype constellations. The Wa, DS-1 and AU-1 genogroups are described as G1-P[8]-I1-R1-C1-M1-A1-N1-T1-E1-H1, G2-P[4]-I2-R2-C2-M2-A2-N2-T2-E2-H2, and G3-P[9]-I3-R3-C3-M3-A3-N3-T3-E3-H3, respectively (Matthijnssens et al., 2008a,b; Matthijnssens et al., 2011). Most G1P[8], G3P[8], and G9P[8] viruses possess a Wa-like genotype constellation, whereas most G2P[4] viruses have a DS-1-like genotype constellation (Heiman et al., 2008). However, since 2012, a DS-1-like G1P[8] (G1-P[8]-I2-R2-C2-M2-A2-N2-T2-E2-H2) strain has spread worldwide (Komoto et al., 2015; Fujii et al., 2019; Luchs et al., 2019). The DS-1-like G3P[8] (G3-P[8]-I2-R2-C2-M2-A2-N2-T2-E2-H2) strain, which is a VP7 mono-reassortant strain between the DS-1-like G1P[8] and equine-like G3 strains has spread widely since 2015 (Arana et al., 2016; Cowley et al., 2016; Utsumi et al., 2018; Fujii et al., 2020; Akane et al., 2021). The bovine-like G8P[8] (G8-P[8]-I2-R2-C2-M2-A2-N2-T2-E2-H2) strain has been prevalent since 2014, particularly in Asia (Hoa-Tran et al., 2016; Tacharoenmuang et al., 2016; Kondo et al., 2017). Additionally, since 2018, G9P[8]-E2 (G9-P[8]-I1-R1-C1-M1-A1-N1-T1-E2-H1), an NSP4 mono-reassortant strain, has become prevalent in Japan (Fujii et al., 2020; Fukuda et al., 2022) and China (Liu et al., 2022). In the last decade, several unusual RVA strains have emerged, causing epidemics.

In this study, we performed comprehensive molecular epidemiological research based on nearly complete genome analyses of RVAs isolated in Japan between 2014 and 2019. Notably, in 2015 and 2018, we detected 42 strains of G1P[8]-E2 (G1-P[8]-I1-R1-C1-M1-A1-N1-T1-E2-H1), a novel NSP4 mono-reassortant strain. Consequently, we focused on G1P[8]-E2 strains and conducted a detailed analysis of their genetic evolutionary trends. This is the last comprehensive surveillance data collected before the COVID-19 pandemic and before the initiation of routine RVA vaccination in Japan.

## 2 Materials and methods

### 2.1 Sample collection

As shown in the map in Supplementary Figure S1, surveillance was conducted at 12 sentinel hospitals in Hokkaido Prefecture (Hakodate Municipal Hospital, Hokkaido Medical Center, Iwamizawa Municipal General Hospital, JCHO Sapporo Hokushin Hospital, Japan Red Cross Urakawa Hospital, NTT Medical Center Sapporo, Rumoi City Hospital, Sunagawa City Medical Center, Steel Memorial Muroran Hospital, Takikawa Municipal Hospital, Tomakomai City Hospital, and Yakumo General Hospital) between 2014–2019. Patients under 15 years of age who were admitted to these hospitals with acute gastroenteritis were screened for the presence of the RVA antigen using commercially available immunochromatographic tests. Analyses were performed on RVA-positive stool specimens collected from patients. The samples were suspended in phosphate-buffered saline (at approximately 10%) and stored at −20°C until required for further analyses. All patients or patient guardians from which samples were collected provided their informed consent. The study protocol was approved by the Ethics Committee of Biomedical Science in the National Institute of Infectious Diseases, Japan (approval number: 956).

### 2.2 RNA extraction

Viral RNA was extracted from stool suspensions using a Direct-zol RNA MiniPrep kit (Zymo Research, Irvine, CA, United States) according to the manufacturer’s instructions. Briefly, 240 μL of TRIzol^®^ LS Reagent was added to 80 μL of stool suspension and homogenized by vortexing. After a 5-min incubation at room temperature, 320 μL of ethanol was added, and the mixture was loaded directly onto the provided spin column. The column was centrifuged at 12,000 × *g* for 1 min and washed with prewash and wash buffer. The purified RNA was then eluted with 40 μL of DNase/RNase-free water.

### 2.3 cDNA library and nucleotide sequencing

Next,-generation sequencing was performed as previously described (Dennis et al., 2014; Doan et al., 2016; Fujii et al., 2019). Briefly, a 200-bp fragment library was constructed for each sample using the NEBNext Ultra RNA Library Prep Kit for Illumina v1.2 (New England Biolabs, Ipswich, MA, United States), according to the manufacturer’s instructions. Library purification was performed using Agencourt AMPure XP magnetic beads (Beckman Coulter, Pasadena, CA, United States) according to the manufacturer’s instructions. DNA concentrations were determined on a Qubit 2.0 fluorometer using the Qubit HS DNA Assay (Invitrogen, Carlsbad, CA, United States). A 151-cycle paired-end-read sequencing run was conducted on a MiSeq desktop sequencer (Illumina, San Diego, CA, United States) using the MiSeq Reagent Kit v2 (300 cycles). Sequence data were analyzed using CLC Genomics Workbench Software v7.0.3 (CLC Bio, Aarhus, Denmark), and sequences of representative strains were deposited in the GenBank/EMBL/DDBJ databases under Accession Nos. LC750855–LC752086, LC763123–LC763221.

### 2.4 Genotyping and phylogenetic analysis

Genotypes of the 11 genome segments were determined using the Rotavirus A Genotyping Tool v 0.1 on Rijksinstituut voor Volksgezondheid en Milieu (RIVM: https://www.rivm.nl/mpf/typingtool/rotavirusa/) and Nucleotide BLAST on the National Center for Biotechnology Information (NCBI: https://blast.ncbi.nlm.nih.gov/Blast.cgi). Near-full-length genome sequences were aligned with reference sequences using MEGA 7 software (Tamura et al., 2013) and MAFFT multiple-sequence alignment software, v 7.0 (Katoh et al., 2009). The best substitution models were selected based on the corrected Akaike information criterion value, as implemented in MEGA 7. Phylogenetic trees were constructed using the maximum likelihood estimation method with 1,000 bootstrap replicates. Representative reference sequences detected before 2019 were selected from GenBank for the construction of phylogenetic trees and subsequent evolutionary analysis. Only sequences with lengths >90% for each segment were used. Sequences of almost identical strains (such as strains detected in the same area and same year) were excluded.

### 2.5 Bayesian evolutionary analysis with BEAST

Evolutionary rates and the Times of Most-Recent Common Ancestor (TMRCAs) were calculated by the Bayesian Markov Chain Monte Carlo (MCMC) method implemented in BEAST v1.8.1 (Drummond et al., 2012). The best-substitution models used for BEAST analyses were calculated using the MEGA 7 software. A strict clock and coalescent exponential growth model (Drummond et al., 2002) was employed. MCMC runs were carried out for 200 million generations to achieve convergence, with sampling conducted every 1,000 steps. Convergence was assessed from the effective sample size after a 10% burn-in using the Tracer software v1.6.^1^ Only parameters with an effective sample size of >200 were accepted. Maximum clade credibility (MCC) time-scaled trees were annotated with Treeannotator and viewed using FigTree v1.4.4.^2^

## 3 Results

### 3.1 Genotype distribution in Hokkaido, Japan

Between 2014 and 2019, a total of 489 RVA-positive stool specimens were identified and analyzed using next-generation sequencing. The genotype constellations of these strains were classified into nine patterns (G1P[8] (Wa), G1P[8]-E2, G1P[8] (DS-1), G2P[4] (DS-1), G3P[8] (Wa), G3P[8] (DS-1), G8P[8] (DS-1), G9P[8] (Wa), G9P[8]-E2) (Supplementary Table S1). The genotype distributions for each year are shown in Table 1. In Japan, RVA gastroenteritis cases occur mainly from January to June, with a peak in March and April. The same trend was observed in our present study. The major prevalent genotype differed by year as follows: G8P[8] (DS-1) (37% of that year’s isolates) in 2014, G1P[8] (DS-1) (65%) in 2015, G9P[8] (Wa) (72%) in 2016, G3P[8] (DS-1) (66%) in 2017, G1P[8]-E2 (53%) in 2018, and G9P[8] (Wa) (26%) in 2019. The G2P[4] (DS-1) and G9P[8] (Wa) genotypes were detected continuously in all six years, and the G8P[8] (DS-1) genotype was detected across five years except in 2018. The G1P[8] (DS-1) genotype was prevalent until 2015 but was subsequently (since 2016) replaced by G3P[8] (DS-1), which possesses a similar genetic background. The G1P[8]-E2 and G9P[8]-E2 genotypes are novel and unusual NSP4 mono-reassortant strains. The G1P[8]-E2 genotype initially spread (to a certain extent) in 2015 but was not detected in 2016 or 2017. This genotype subsequently re-emerged in 2018 but was not detected in 2019. In contrast, the G9P[8]-E2 genotype was continuously detected in 2018 and 2019. The genotype distribution for each hospital is shown in Supplementary Figure S2.

**Table 1:** RVA genotype distributions in Hokkaido, Japan (2014–2019).

Type	2014		2015		2016		2017		2018		2019	
	No.	(%)	No.	(%)	No.	(%)	No.	(%)	No.	(%)	No.	(%)
G1P[8] (Wa)	13	(15%)	3	(3%)			1	(2%)			10	(21%)
G1P[8]-E2			11	(10%)					31	(53%)		
G1P[8] (DS-1)	8	(9%)	71	(65%)								
G2P[4] (DS-1)	15	(17%)	3	(3%)	8	(6%)	8	(14%)	15	(25%)	3	(6%)
G3P[8] (Wa)									2	(3%)		
G3P[8] (DS-1)					25	(20%)	39	(66%)	1	(2%)	2	(4%)
G8P[8] (DS-1)	32	(37%)	5	(5%)	2	(2%)	6	(10%)			9	(19%)
G9P[8] (Wa)	19	(22%)	17	(15%)	92	(72%)	5	(8%)	8	(14%)	12	(26%)
G9P[8]-E2									2	(3%)	11	(23%)
Total No.	87	(100%)	110	(100%)	127	(100%)	59	(100%)	59	(100%)	47	(100%)

### 3.2 Phylogenetic tree of VP7 gene in G1 strains

**Figure 1:**
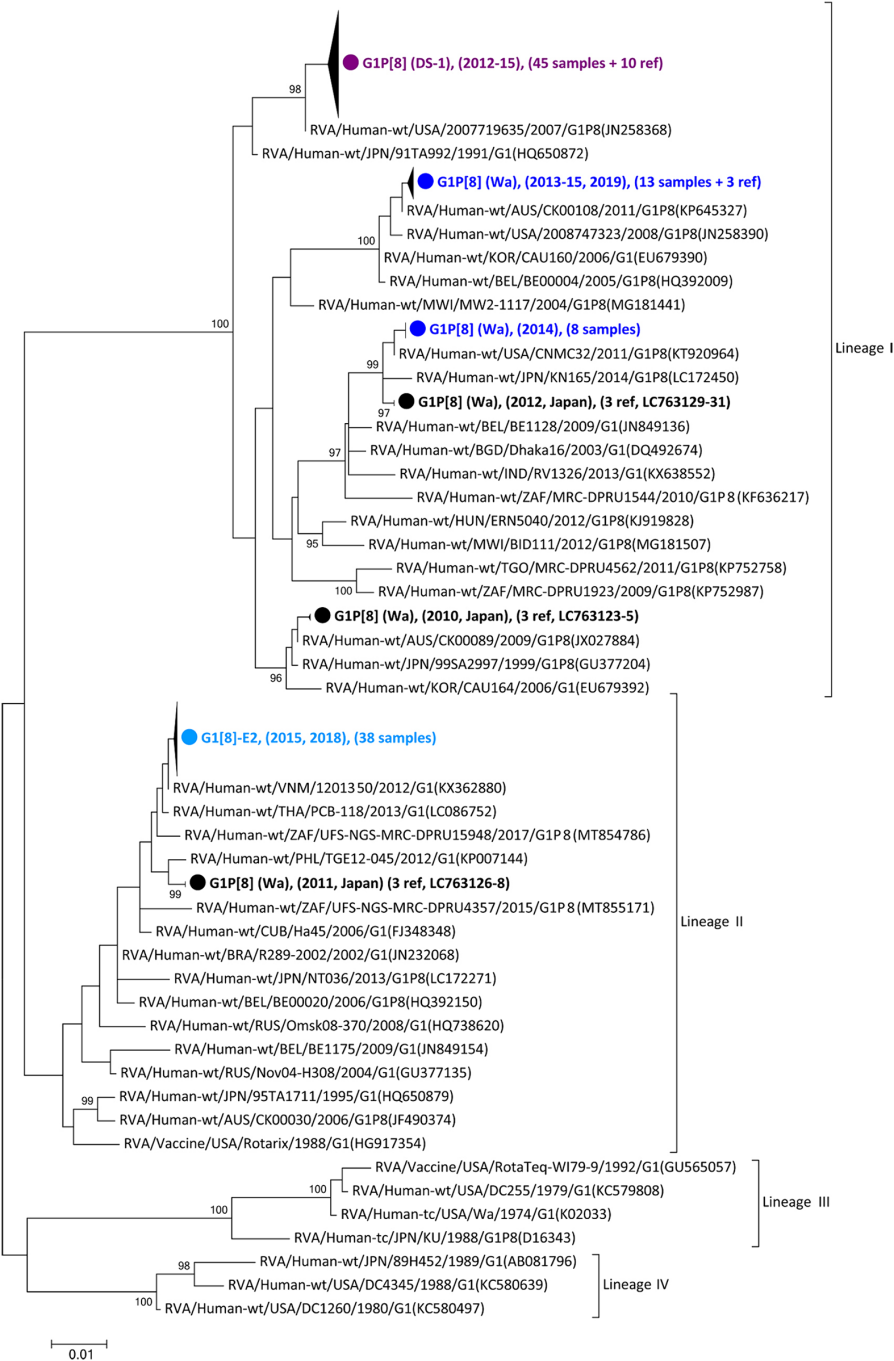
Phylogenetic tree of the G1-type VP7 gene. The tree was constructed from the nucleotide sequences of 168 G1 strains (104 detected in this study, 26 corresponding to other recent Japanese strains; and 38 consisting of representative foreign strains, including vaccine strains). The strains detected in this study were grouped into four confined clusters that are shown here as triangles and colored according to the group: blue, G1P[8] (Wa); purple, G1P[8] (DS-1); light blue, G1P[8]-E2 strains. The number of compressed strains is shown in parentheses, with “ref” indicating reference strains. Closed circles indicate compressed representative strains that were detected in Japan between 2010 and 2012 (Accession Nos. LC763123 – LC763131). The phylogenetic trees were constructed using the maximum-likelihood estimation method with 1,000 bootstrap replicates in MEGA 7 software. Percent bootstrap support is indicated by the value at each node when the value is 95% or larger.

As the first step in clarifying the relationship between the new NSP4 mono-reassortant G1P[8]-E2 strain and other major G1 strains, a phylogenetic tree of the G1-type VP7 gene segment was constructed (Figure 1). This tree incorporated sequences from 168 strains, including the 99 detected in this study, 25 other recent Japanese strains, and 44 representative foreign strains (including the vaccine strains). Recent Japanese G1 strains were divided into Lineages I and II, corresponding to strains that were considered major and minor, respectively, in Japan. All the G1P[8]-E2 strains belonged to Lineage II and were concentrated in a single cluster. The differences among the G1P[8]-E2 strains isolated in 2015 and 2018 were minute, with nucleotide identities exceeding 99.8%. The VP7 gene segments of these G1P[8]-E2 strains were genetically similar to those of G1P[8] (Wa) strains detected in 2011 in Japan (>98.5% sequence identity). Lineage I strains detected in this study were divided into three clusters. All the G1P[8] (DS-1) strains were concentrated in one cluster. The G1P[8] (Wa) strains detected in 2019 belonged to the same cluster as the related strains detected from 2013–2015. Furthermore, the G1P[8] (Wa) strains detected in 2014 were genetically similar to those detected in 2012.

### 3.3 Evolutionary rate of RVA genome

Next, to investigate the evolutionary history of the Japanese RVA strains, the evolutionary rate was calculated for each genotype of each gene (Supplementary Table S2). The number of sample sequences used for the calculation and best substitution models are shown in Supplementary Table S2. For this analysis, only human wild-type RVA strains were used to calculate the natural rate of evolution in the human population. Vaccine, tissue-cultured, and animal-derived strains were excluded. The estimated mean evolutionary rates ranged between 5.48 × 10^−4^ (R1-type VP1) – 1.18 × 10^−3^ (E2-type NSP4) nucleotide substitutions per site per year. The rates in genotype-2 (i.e., DS-1-like genotypes) were higher than those of genotype-1 (i.e., Wa-like genotypes) in many genes (VP6, VP1, VP2, VP3, NSP2, NSP3, NSP4, and NSP5).

### 3.4 Time-scaled MCC trees

**Figure 2:**
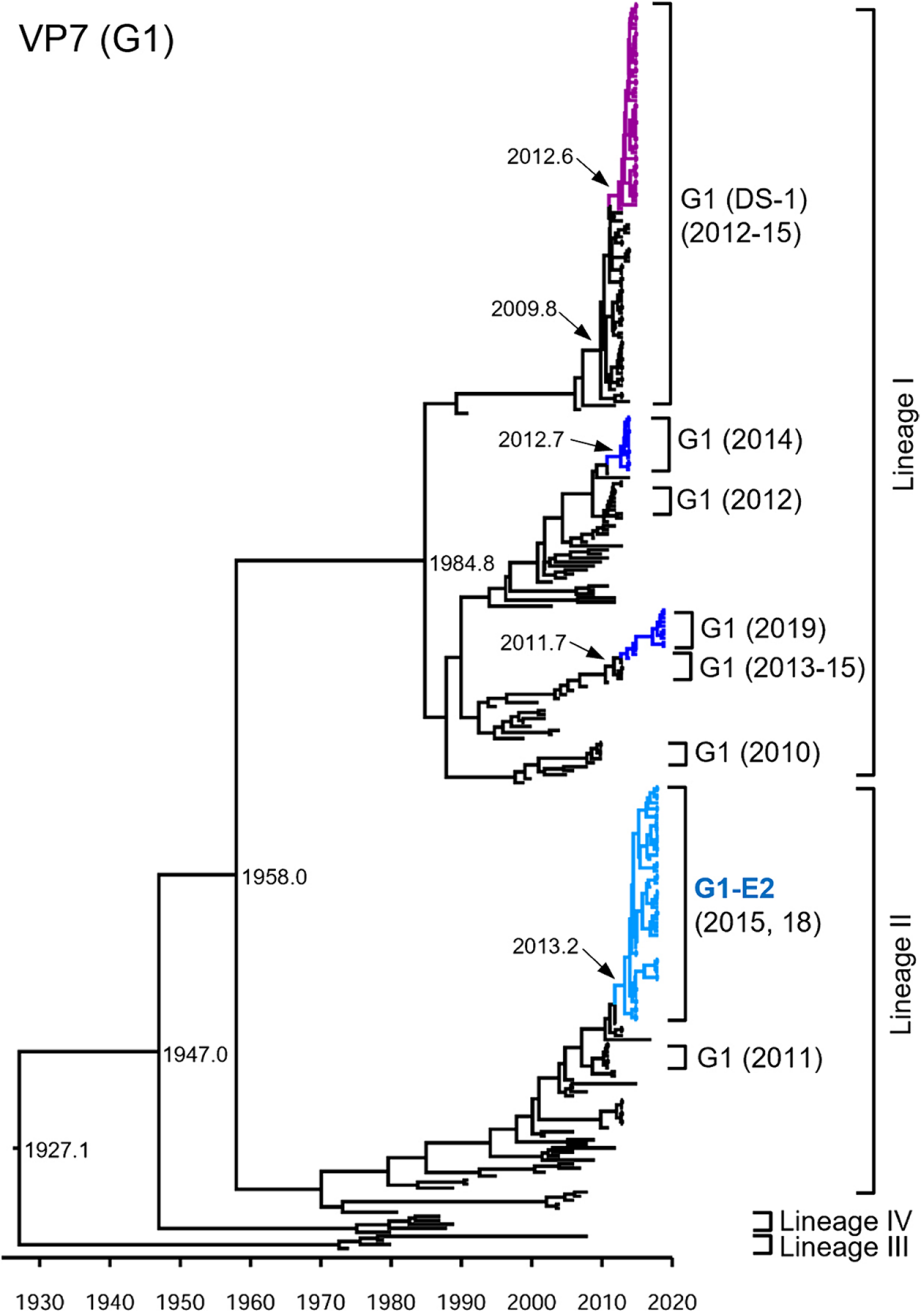
Maximum clade credibility (MCC) tree of the VP7 gene. Bayesian time-scaled tree for the G1-type VP7 gene was constructed. The representative Japanese strain groups are indicated by square brackets, and their type and year of detection are shown on the right. Estimated ages are indicated with arrows for representative nodes. The strains detected in this study are colored according to the group: blue, G1P[8] (Wa); purple, G1P[8] (DS-1); light blue, G1P[8]-E2.

Focusing on the G1P[8]-E2 strains, the time-scaled MCC trees were constructed for each genotype for each gene. The numbers of sequences and models used for each tree are listed in Supplementary Table S2. In the VP7 G1 MCC tree (Figure 2), the Japanese G1 strains detected in this study were concentrated into four clusters, as shown in the phylogenetic tree (Figure 1). The G1P[8] strains detected in Japan between 2011–2019 were sorted into different clusters each year, indicating that the same strains were unlikely to be continuously prevalent in a given region. However, all the detected G1P[8]-E2 strains shared a recent common ancestor according to the TMRCAs of 2013.2 (95% highest posterior density (HPD) interval: 2012.1–2014.2) (Figure 2; Supplementary Table S3).

**Figure 3:**
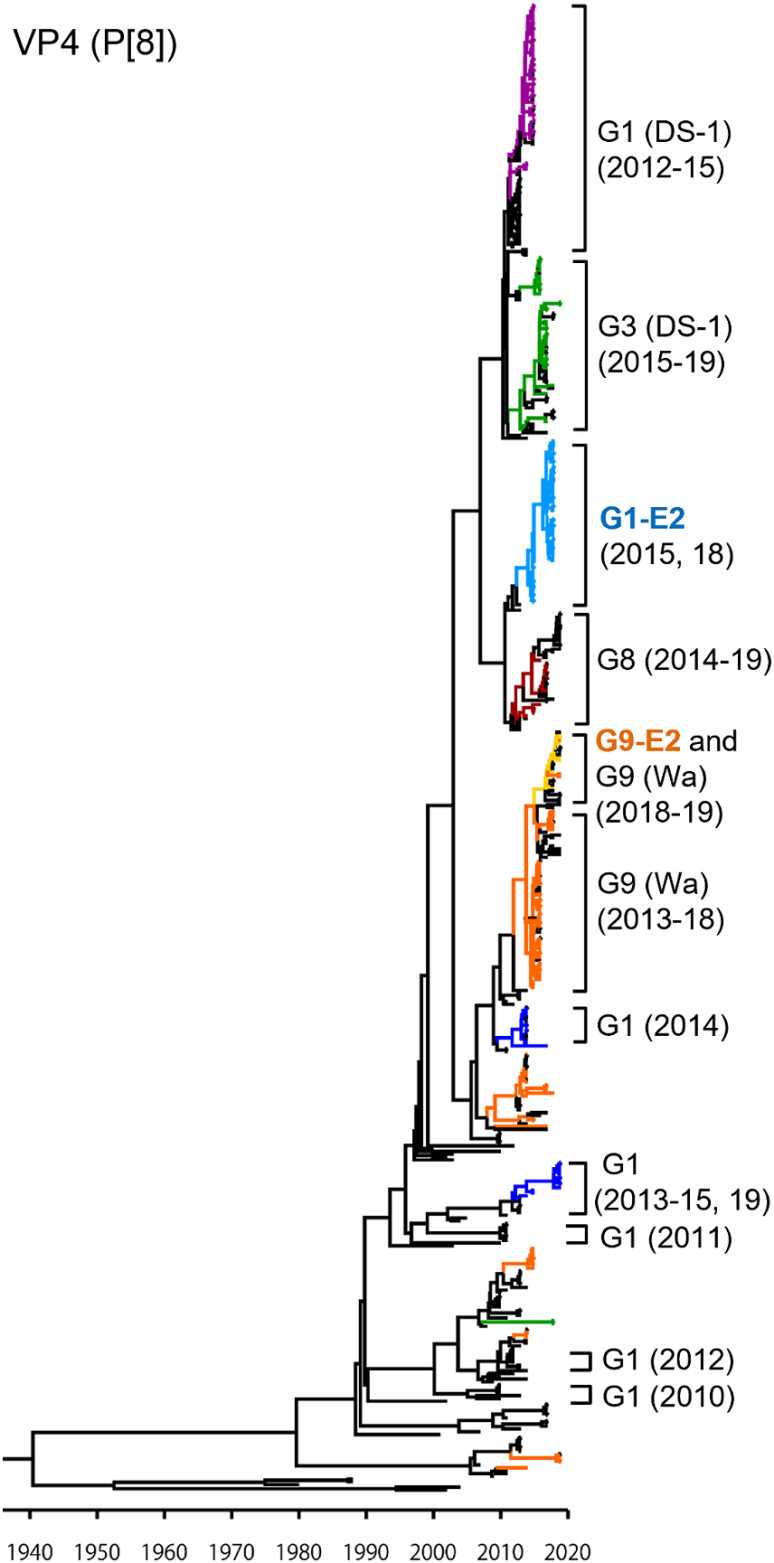
Maximum clade credibility (MCC) tree of the VP4 gene. Bayesian time-scaled tree for the P[8]-type VP4 gene was constructed. The representative Japanese strain groups are indicated by square brackets, and their type and year of detection are shown on the right. Estimated ages are indicated with arrows for representative nodes. The strains detected in this study are colored according to the group: blue, G1P[8] (Wa); purple, G1P[8] (DS-1); light blue, G1P[8]-E2; green, G3P[8] (Wa and DS-1); brown, G8P[8]; orange, G9P[8] (Wa); and yellow, G9P[8]-E2.

As presented in the VP4 P[8] MCC tree (Figure 3), the G1P[8] (DS-1), G3P[8] (DS-1), G1P[8]-E2, and G8P[8] (DS-1) strains, which recently emerged as reassortant viruses, were segregated into their own independent clusters. In contrast, traditional viruses (G1P[8] (Wa), G3P[8] (Wa), and G9P[8] (Wa)) that have been prevalent for a long time were distributed in multiple clusters, indicating that these viruses have undergone complex evolution over a long period. The G9P[8]-E2 strains were concentrated in one cluster but intermixed with a few of the G9P[8] (Wa) strains. Similar to VP7, the VP4 genes of the Japanese G1P[8] strains were sorted into different clusters from year to year. The TMRCAs of VP4 genes of the G1P[8]-E2 and the G9P[8]-E2 strains were estimated as 2013.7 (95% HPD, 2005.1–2014.7) and 2016.3 (95% HPD, 2004.1–2017.2), respectively (Figure 3; Supplementary Table S3). It appears that the VP4 genes of the G1P[8]-E2 strains have a common ancestor with those of the G8P[8] strains.

**Figure 4:**
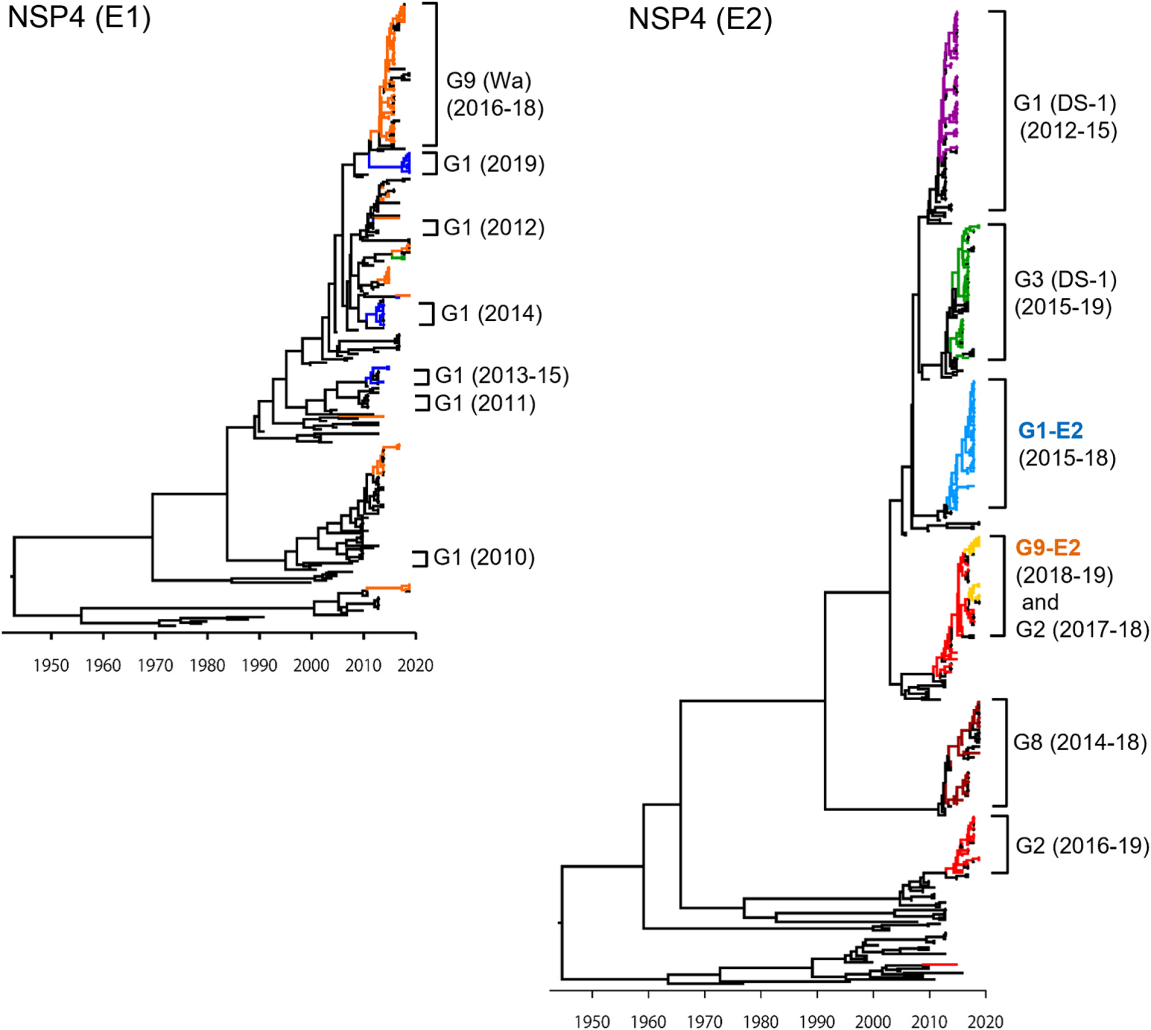
Maximum clade credibility (MCC) trees of the NSP4 gene. Bayesian time-scaled trees for the NSP4 genes (E1 type and E2 type) were constructed. The representative Japanese strain groups are indicated by square brackets, and their type and year of detection are shown on the right. Estimated ages are indicated with arrows for representative nodes. The strains detected in this study are colored according to the group: blue, G1P[8] (Wa); purple, G1P[8] (DS-1); light blue, G1P[8]-E2; red, G2P[4]; green, G3P[8] (Wa and DS-1); brown, G8P[8]; orange, G9P[8] (Wa); and yellow, G9P[8]-E2.

In the NSP4 MCC tree (Figure 4), as with the VP4 tree, the G1P[8] (DS-1), G3P[8] (DS-1), G1P[8]-E2, and G8P[8] (DS-1) strains each segregated into their own independent clusters (E2 tree); the G1P[8] (Wa), G3P[8] (Wa), and G9P[8] (Wa) strains were distributed among multiple clusters (E1 tree). Similar to VP7 and VP4 genes, Japanese G1P[8] (Wa) strains belonged to different clusters from year to year (E1 tree). In VP7 and VP4 trees (Figures 2, 3, respectively), the G1P[8] (Wa) (2019) and the G1P[8] (Wa) (2013–2015) strains belonged to one cluster; in contrast, in the NSP4 (E1) tree (Figure 4), the G1P[8] (Wa) (2019) strains were sorted as a separate cluster from the G1P[8] (Wa) (2013–2015) strains and appeared to have a common ancestor with the G9P[8] (Wa) (2016–2018) strains. The G9P[8]-E2 strains were segregated into one cluster but were intermixed with a few of the G2P[4] (DS-1) strains (E2 tree). The TMRCAs of the G1P[8]-E2 and G9P[8]-E2 strains were estimated as 2013.0 (95% HPD, 2012.8–2014.2) and 2014.4 (95% HPD, 2014.2–2015.5), respectively (Figure 4; Supplementary Table S3).

**Figure 5:**
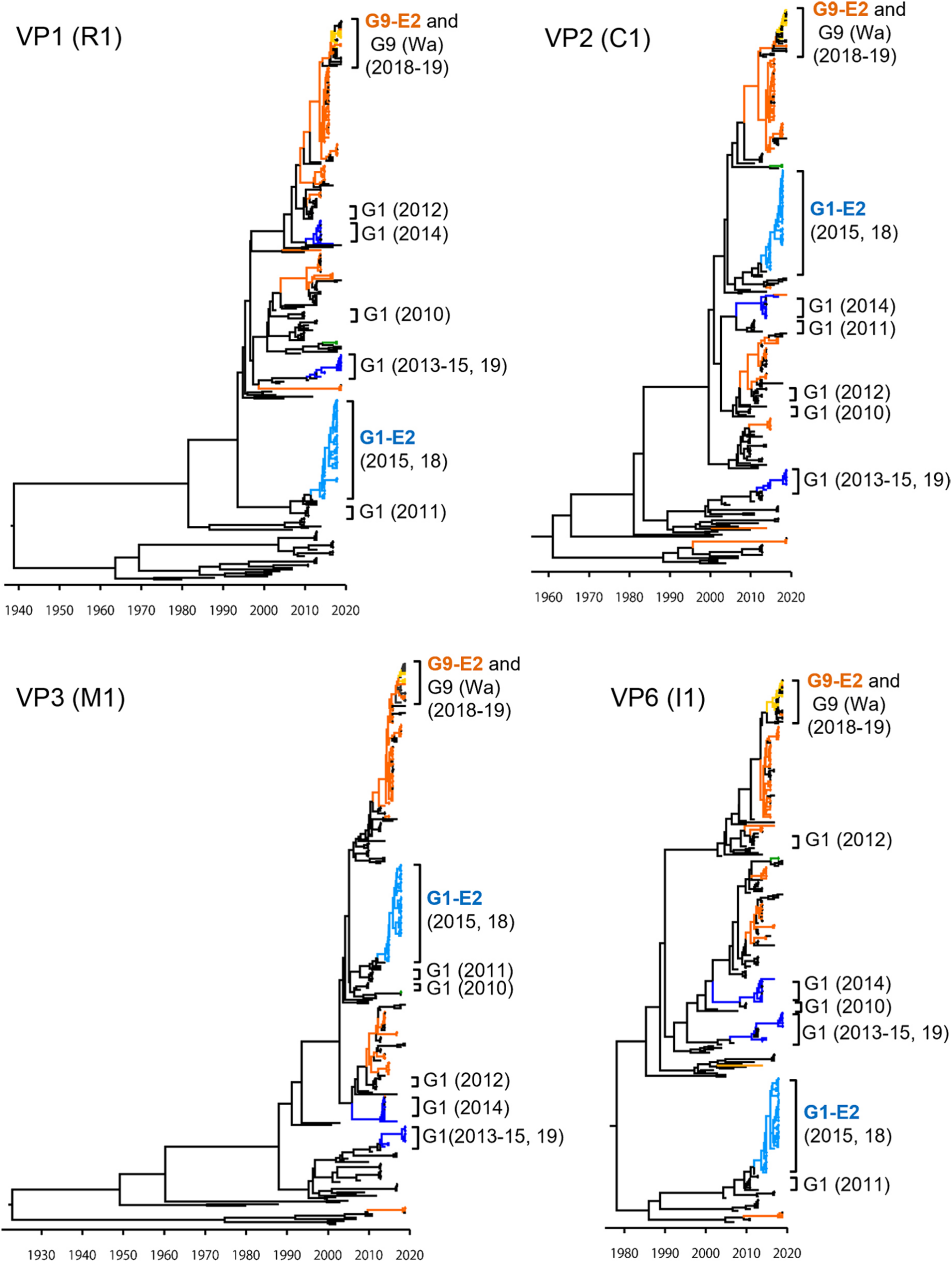
Maximum clade credibility (MCC) trees of other VP genes. Bayesian time-scaled trees for the VP1 (R1), VP2 (C1), VP3 (M1), and VP6 (I1) genes were constructed. The representative Japanese strain groups are indicated by square brackets, and their type and year of detection are shown on the right. The strains detected in this study are colored according to the group: blue, G1P[8] (Wa); light blue, G1P[8]-E2; green, G3P[8] (Wa); orange, G9P[8] (Wa); and yellow, G9P[8]-E2.

**Figure 6:**
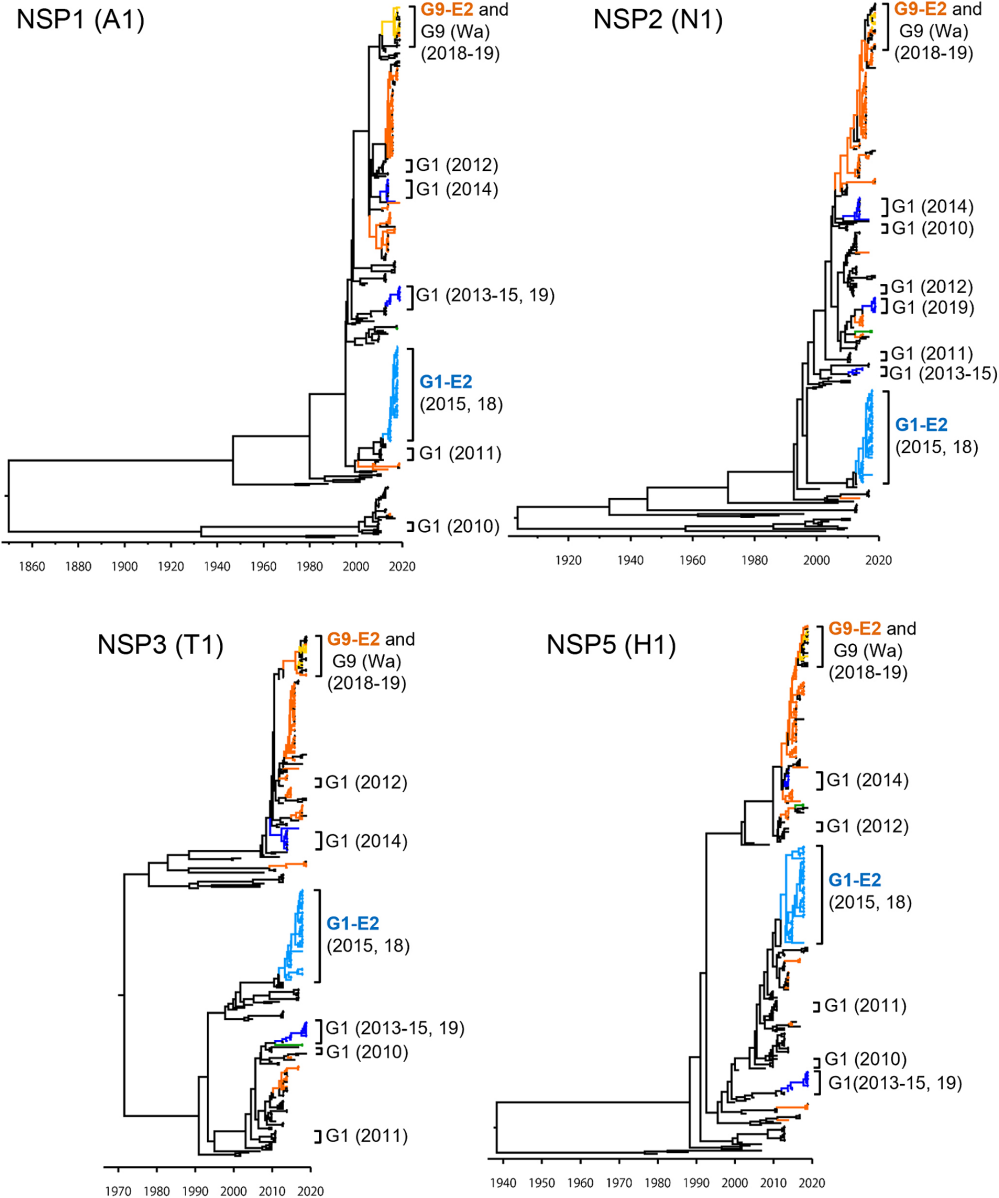
Maximum clade credibility (MCC) trees of other NSP genes. Bayesian time-scaled trees for the NSP1 (A1), NSP2 (N1), NSP3 (T1), and NSP5 (H1) genes were constructed. The representative Japanese strain groups are indicated by square brackets, and their type and year of detection are shown on the right. The strains detected in this study are colored according to the group: blue, G1P[8] (Wa); light blue, G1P[8]-E2; green, G3P[8] (Wa); orange, G9P[8] (Wa); and yellow, G9P[8]-E2.

To investigate the derivation of the G1P[8]-E2 strains and the evolutionary history of Japanese G1P[8] strains, MCC trees were constructed for VP1 (R1), VP2 (C1), VP3 (M1), VP6 (I1), NSP1 (A1), NSP2 (N1), NSP3 (T1), and NSP5 (H1) genes (Figures 5, 6). These MCC trees generally showed common trends: the G1P[8]-E2 and G9P[8]-E2 strains segregated into separate clusters, whereas the G9P[8]-E2 strains intermixed with a few G9P[8] (Wa) strains. Additionally, the Japanese G1P[8] (Wa) strains were sorted into different clusters from year to year. However, the NSP2 tree differed from the others; the G1P[8] (Wa) (2019) clustered strains were separated from those of the G1P[8] (Wa) (2013–2015) strains (similar to the NSP4 (E1) tree). The NSP2 genes in the G1P[8] (Wa) (2019) strain were similar to those in the G9P[8] (Wa) strain detected in 2015. Together, these data indicated that the G1P[8] (Wa) (2019) strains were derived from the G1P[8] (Wa) (2013–2015) strains, but two genes, NSP2 and NSP4, appeared to have been derived from other strains (probably the G9P[8] strains) by intra-genogroup reassortment.

### 3.5 The derivations of NSP4 mono-reassortant strains

Two newly emerged NSP4 mono-reassortant strains, G1P[8]-E2 and G9P[8]-E2, are prevalent in Hokkaido, Japan. The genotype constellations were the same, except for VP7 (Supplementary Table S1). Therefore, Nucleotide BLAST was used to search databases for strains that showed the strongest similarity for each gene (Supplementary Table S3). The G1P[8]-E2 strains demonstrated the highest homology to Asian strains (Vietnam or Thailand), with nucleotide identities exceeding 99.5%, whereas the G9P[8]-E2 strains demonstrated the highest homology to the Japanese strains obtained in this study. The TMRCA for the G1P[8]-E2 and G9P[8]-E2 strains ranged from 2012.9 to 2013.7 and 2014.4 to 2016.5, respectively. These results suggest that G1P[8]-E2 and G9P[8]-E2 were generated from distinct independent inter-genogroup reassortment events.

### 3.6 Amino acid sequences of VP7 epitope

**Figure 7:**
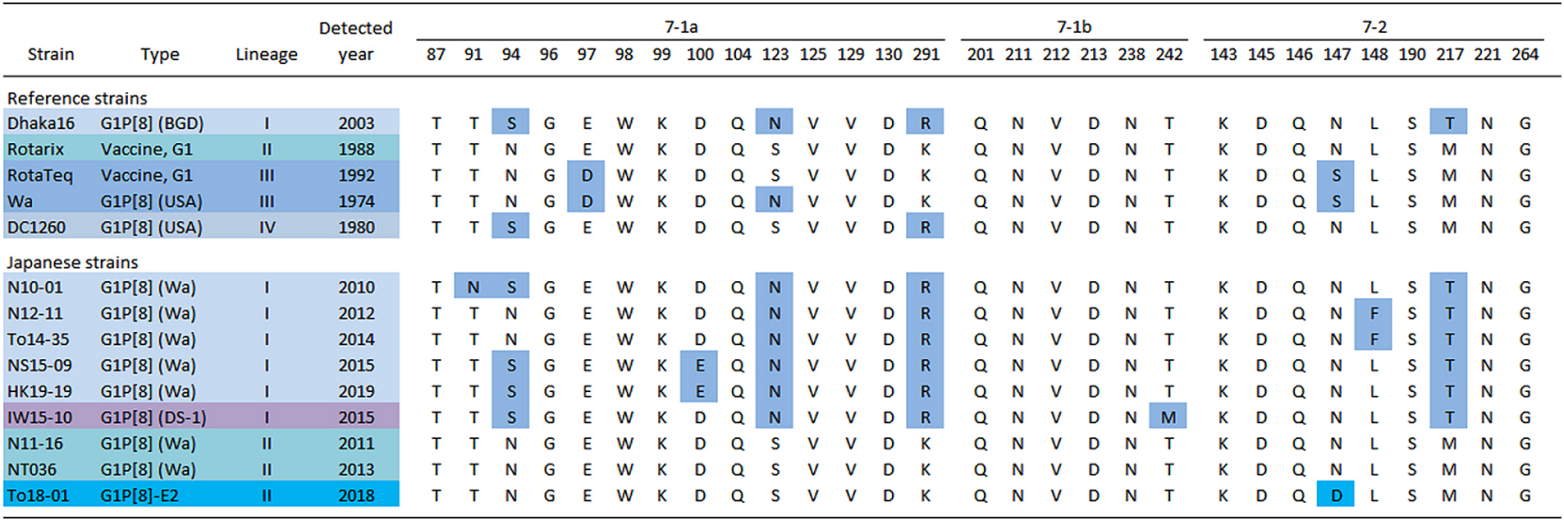
Alignment of antigenic residues in the VP7 protein. The amino acid sequences of VP7 antigenic epitopes (7-1a, 7-1b, and 7–2) of representative G1 strains detected in Japan are shown. Amino acids that differ from those encoded by the Rotarix vaccine strain are colored in blue [accession nos. Dhaka16: DQ492674, Rotarix: HG917354, RotaTeq (WI79-9): GU565057, Wa: K02033, DC1260: KC580497, N10-01: LC763123, N12-11: LC763129, To14-35: LC105369, NS15-09: LC750905, HK19-19: LC750883, IW15-10: LC750890, N11-16: LC763126, NT036: LC172271 and To18-01: LC750937].

To investigate the cause of the discontinuity in the G1 epidemic strains, the amino acid sequences of the VP7 epitopes of representative Japanese strains from each year were compared (Figure 7) (Aoki et al., 2009). The analysis revealed that these strains had several patterns with different epitope sequences. Compared to the Rotarix vaccine strains, the Japanese G1 Lineage I strains commonly carry the S123N, K291R, and M217T mutations. Additionally, the strains detected in 2012 and 2014 harbored the L148F mutation, whereas the strains detected in 2015 and 2019 harbored the N94S and D100E mutations. The G1P[8] (DS-1) strain isolated from 2012 to 2015 had N94S and T242M mutations. The Japanese G1 Lineage II strains from 2011 and 2013 did not exhibit any mutations compared with the Rotarix vaccine strain. However, the G1P[8]-E2 strains from 2015 and 2018 carried the characteristic N147D mutation.

## 4 Discussion

Our study analyzed the nearly complete genome sequences of RVA strains detected in Hokkaido, Japan, over the course of six years (2014–2019) and assessed their genetic relationships with other domestic and foreign RVA isolates. This work, in combination with our previous studies, has provided several useful insights into recent trends in Japanese RVA strains. Recently, several unusual strains including (G1P[8] (DS-1), G3P[8] (DS-1), G8P[8] (DS-1), and G9P[8]-E2) have emerged in Japan (Table 1; Supplementary Table S1). Each of these novel strains achieved nationwide prevalence for 2–3 years, after which each strain was detected in several smaller outbreaks, as reported in the Infectious Agents Surveillance Report^3^ and previous studies (Fujii et al., 2019, 2020; Tsugawa et al., 2021). However, G1P[8] (DS-1) strains have become almost undetectable in recent years and have been replaced by equine-like G3P[8] (DS-1) strains with a similar genetic background. In contrast, G2P[4] and G9P[8] strains remained prevalent in Japan throughout the study period. G8P[8] (DS-1) strains were continuously detected since 2014 (except for 2018). However, the recent emergence of two classes of NSP4 mono-reassortant strains, G1P[8]-E2 and G9P[8]-E2, is remarkable. The G9P[8]-E2 strains are widely prevalent in Japan (Fujii et al., 2020; Fukuda et al., 2022). Phan et al. reported the detection of a single G1P[8]-E2 strain in Hokkaido, Japan, in 2018 (Phan et al., 2020), but only limited data were provided. This is the first study that provides evidence that the G1P[8]-E2 strain caused large-scale epidemics in Hokkaido, Japan in 2015 and 2018. The G1P[8]-E2 strains were detected in Tomakomai, Muroran, and Iwamizawa in 2015, as well as in Tomakomai, Muroran, Iwamizawa, Sapporo, Hakodate, Sunagawa, and Urakawa in 2018. However, no G1P[8]-E2 strains were detected in any part of Hokkaido in 2016 or 2017, and to our knowledge, none have been detected outside Hokkaido to date. In Japan, RVA strains similar to those found in China and Southeast Asia are often detected. On the other hand, the genotype distribution seems to vary from country to country, with G9P[8] continuing to be the major prevalent strain in China (Tian et al., 2021), and G4P[6] and G8P[6] being prevalent in Korea (Lee et al., 2018). Further studied are required to understand the causes of these differences.

The observation of prevalent G1P[8]-E2 strains in discontinuous years is interesting. However, it remains unknown where this virus was present during the intervening two-year interval. This virus could have been maintained among older children, adults, and vaccinators with asymptomatic infections, as suggested by several studies (Gunawan et al., 2019; Bennett et al., 2021). The discontinuity in the prevalence of these epidemic strains may be attributed to developed herd immunity. Most patients with RVA gastroenteritis are infants; once a particular strain is prevalent in an area, many children acquire immunity to that strain. Consequently, other strains with different antigenicities gain prevalence in the following season(s). As generations shift, immunity to the previous strain decreases, and other strains become more prevalent. The same mechanism may be responsible for the annual changes in the major epidemic strains observed in this study (G8P[8] in 2014, G1P[8] (DS-1) in 2015, G9P[8] (Wa) in 2016, G3P[8] (DS-1) in 2017, G1P[8]-E2 in 2018, and G9P[8] (Wa) in 2019) (Table 1). In Japan, RVA vaccines were introduced in November 2011 and were incorporated into routine vaccination programs in October 2020. The vaccination rate during the study period was estimated to be approximately 50–70% (Tsugawa et al., 2021). Therefore, the prevalence of RVA strains in Japan reflects a complex situation involving two major factors: herd immunity acquired by vaccination and natural infection with wild strains. Further studies are needed to clarify the differences in the quality of cross-reactivity among immune responses acquired by vaccination and natural infection with wild strains. While previous studies have elucidated upon the development of herd immunity induced by RNA vaccines (Paulke-Korinek et al., 2011; Mast et al., 2015; Ito and Higashigawa, 2021), the G12 strain continued to be predominant in the United States even after vaccine introduction. It has been suggested that one reason may be due to a lower level of vaccine efficacy against G12 strains (i.e., different VP7 genotypes) (Esona et al., 2021). Although difficult to evaluate, Ianiro et al. (2016) also noted that the genetic shift of G1 strains in Italy could be attributed herd immunity.

In this study, G1 RVA was detected each year, except in 2016, and phylogenetic analyses showed that recent G1 strains were segregated into many clusters (Figures 1, 2). The prevalent strains were sorted into a single cluster in 2018 and 2019, however, in 2014 and 2015, they were sorted into multiple clusters. As the years progressed, epidemic strains were often replaced by strains from other clusters. This observation suggests that epidemic strains may shift in response to the effects of herd immunity, even among strains of a specific genotype. The data obtained by comparing VP7 epitope sequences (Figure 7) support this hypothesis. Previous studies have shown that amino acid substitutions at positions 94, 97, 147, and 291 of the VP7 gene significantly affect the antigenic recognition of human G1 strains (Coulson and Kirkwood, 1991). Compared to the vaccine strains, the Japanese G1 strains have several mutations. Mutation patterns differed depending on the year of detection. Mutations N94S, S123N, M217T, and K291R have been reported in strains isolated from other countries (Novikova et al., 2020; Zhou et al., 2020), suggesting that these substitutions have already been reported in recent G1 epidemic strains. Additionally, in this study, the VP7 gene harbored the L148F mutation in 2012 and 2014, the D100E mutation in 2015 and 2019, the T242M mutation in the G1P[8] (DS-1) strain in 2012–2015, and the N147D mutation in the G1P[8]-E2 strain in 2015 and 2018. These substitutions may be responsible for immune escape and annual changes in the prevalent strains. Mwangi et al. previously reported the presence of the N147D mutation in South African strains following vaccine introduction and showed that this substitution destabilized the structure of the VP7 protein (Mwangi et al., 2020). However, strains with the same mutations were observed in Thailand (LC086752) and Vietnam (KX362880) and were closely related to the G1P[8]-E2 strains detected in this study (Figure 1). Thus, it is likely that viruses encoding VP7 with the N147D substitution retain sufficient stability to spread. Therefore, these South African, Thai, and Vietnamese strains are thought to be derived from the same parental strain as the G1P[8]-E2 strain, but all exhibit the G1P[8]-E1 genotype constellation. Consequently, the inter-genogroup reassortment event of the NSP4 gene (G1P[8]-E1 to G1P[8]-E2) is hypothesized to have occurred after the introduction of the N147D substitution, estimated to have occurred approximately in 2013 (Supplementary Table S3). The epitope sites shown here were identified using a limited number of strains. Epitopes that have not yet been identified may also be involved in antigenicity; however further studies are required to clarify this.

In this study, the evolutionary rate of each gene was calculated for each genotype (Supplementary Table S2), and the resulting values ranged from 5.48 × 10^−4^ (for R1-type VP1) to 1.18 × 10^−3^ (for E2-type NSP4) /site/year. For eight genes (VP1, VP2, VP3, VP6, NSP2, NSP3, NSP4, and NSP5), the evolutionary rates tended to be higher in genotype-2 viruses than in genotype-1. Similar trends were reported in other studies (Liu et al., 2022). This difference may reflect high sequence diversity due to the emergence and prevalence of new DS-1-like strains in recent years. Previous studies have suggested that reassortment frequently occurs in the NSP4 segment (Agbemabiese et al., 2016; Fujii et al., 2019). The emergence of G1P[8]-E2 and G9P[8]-E2 may be attributed to the characteristics of the NSP4 segment, which is easily substitutable, and the increasing presence of strains bearing DS-1-like genotypes. NSP4 is a known multifunctional protein associated with enterotoxin activity, RNA replication, interaction with viroplasm, intracellular calcium regulation, and pathogenicity (Estes and Greenberg, 2013). NSP4 is a potential target for protective immunity, but its proportion in the total immune response to RVA is unknown. Since the E2 type of the NSP4 gene is also carried by conventionally prevalent G2, G8, and equine-like G3 strains, it is unlikely that the emergence of G1P[8]-E2 alone will dramatically change the quality of herd immunity.

This study demonstrates the complexity of RVA epidemic strains in Japan. Because many new reassortant strains have emerged in recent years, it has become increasingly difficult to predict which strains will be prevalent in the future. Our findings will be useful in predicting future epidemic strains in areas where RVA remains prevalent. Since the SARS-CoV-2 pandemic in 2020, the outbreak of RVA has dramatically decreased in Japan, as reported in the Infectious Diseases Weekly Report (IDWR; https://www.niid.go.jp/niid/en) and previous studies (Fukuda et al., 2021; Hibiya et al., 2022). This shift is considered to have been due to travel, social restrictions, and non-pharmaceutical interventions (Dapper et al., 2022). After resuming social activities, RVA strains are speculated to once again readily spread domestically and internationally, as noted in other articles (Lappe et al., 2023; Wang et al., 2023). Continuous molecular epidemiological studies based on full genome analysis will continue to be critical for detecting the emergence of new epidemic RVA strains. Moreover, it is important to study the cross-reactivity of the protective immune responses against recent epidemics.

## Data availability statement

The datasets presented in this study can be found in online repositories. The names of the repository/repositories and accession number(s) can be found at: https://www.ncbi.nlm.nih.gov/genbank/, LC750855–LC752086, LC763123–LC763221.

## Ethics statement

The studies involving humans were approved by the study protocol was approved by the Ethics Committee of Biomedical Science in the National Institute of Infectious Diseases, Japan (approval number: 956). The studies were conducted in accordance with the local legislation and institutional requirements. Written informed consent for participation in this study was provided by the participants’ legal guardians/next of kin.

## Author contributions

YoF: Conceptualization, Data curation, Formal analysis, Funding acquisition, Investigation, Methodology, Project administration, Resources, Software, Supervision, Validation, Visualization, Writing – original draft, Writing – review & editing. TaT: Conceptualization, Data curation, Formal analysis, Investigation, Methodology, Project administration, Resources, Supervision, Writing – review & editing. YuF: Data curation, Resources, Writing – review & editing. SA: Data curation, Resources, Writing – review & editing. SH: Data curation, Resources, Writing – review & editing. YA: Data curation, Resources, Writing – review & editing. KK: Data curation, Resources, Writing – review & editing. YS: Data curation, Resources, Writing – review & editing. ToT: Data curation, Resources, Writing – review & editing. TS: Data curation, Resources, Writing – review & editing. YH: Data curation, Resources, Writing – review & editing. NK: Data curation, Resources, Writing – review & editing. TM: Data curation, Resources, Writing – review & editing. SK: Data curation, Resources, Writing – review & editing. RH: Data curation, Resources, Writing – review & editing. MK: Data curation, Resources, Writing – review & editing. YT: Data curation, Resources, Writing – review & editing. KN: Data curation, Resources, Writing – review & editing. TO: Data curation, Resources, Writing – review & editing. MY: Data curation, Resources, Writing – review & editing. SN: Data curation, Resources, Writing – review & editing. AN: Data curation, Investigation, Project administration, Resources, Writing – review & editing. WK: Data curation, Resources, Writing – review & editing. HH: Data curation, Project administration, Resources, Writing – review & editing. ST-H: Data curation, Resources, Writing – review & editing. MF: Data curation, Resources, Writing – review & editing. MM: Conceptualization, Funding acquisition, Project administration, Supervision, Writing – review & editing.

## Footnotes

^1^ http://tree.bio.ed.ac.uk/software/tracer/

^2^ http://tree.bio.ed.ac.uk/software/figtree/

^3^ https://www.niid.go.jp/niid/en/iasr.html

## Supplementary material

Supplementary Figure S1 A map showing the location of the hospitals where the surveillance was conducted. Three hospitals are located in Sapporo City, namely, NTT Medical Center Sapporo (NTT), JCHO Sapporo Hokushin Hospital (JSH), and Hokkaido Medical Center (HMC). Other hospitals are indicated by city name.

Supplementary Figure S2 RVA genotype distributions for each hospital (2014–2019). Three hospitals, namely, NTT Medical Center Sapporo (NTT), JCHO Sapporo Hokushin Hospital (JSH), and Hokkaido Medical Center (HMC), are located in Sapporo city. Other hospitals are indicated by city name.

Supplementary Table S1 Genotype constellations of RVA strains detected in Hokkaido, Japan (2014–19).

Supplementary Table S2 Evolutionary rate of each gene of rotaviruses.

Supplementary Table S3 The Times of Most-Recent Common Ancestor (TMRCA) of NSP4 mono-reassortant strains.
