# Supplementary material for: Molecular evolutionary analysis of novel NSP4 mono-reassortant G1P[8]-E2 rotavirus strains that caused a discontinuous epidemic in Japan in 2015 and 2018
Source: Front Microbiol. 2024 Jul 10;15:1430557. doi: 10.3389/fmicb.2024.1430557 (PMC11266183; doi:10.3389/fmicb.2024.1430557)
Supplement: Supplementary Figure S1 — A map showing the location of the hospitals where the surveillance was conducted. Three hospitals are located in Sapporo City, namely, NTT Medical Center Sapporo (NTT), JCHO Sapporo Hokushin Hospital (JSH), and Hokkaido Medical Center (HMC). Other hospitals are indicated by city name. [file Image_1.pdf]

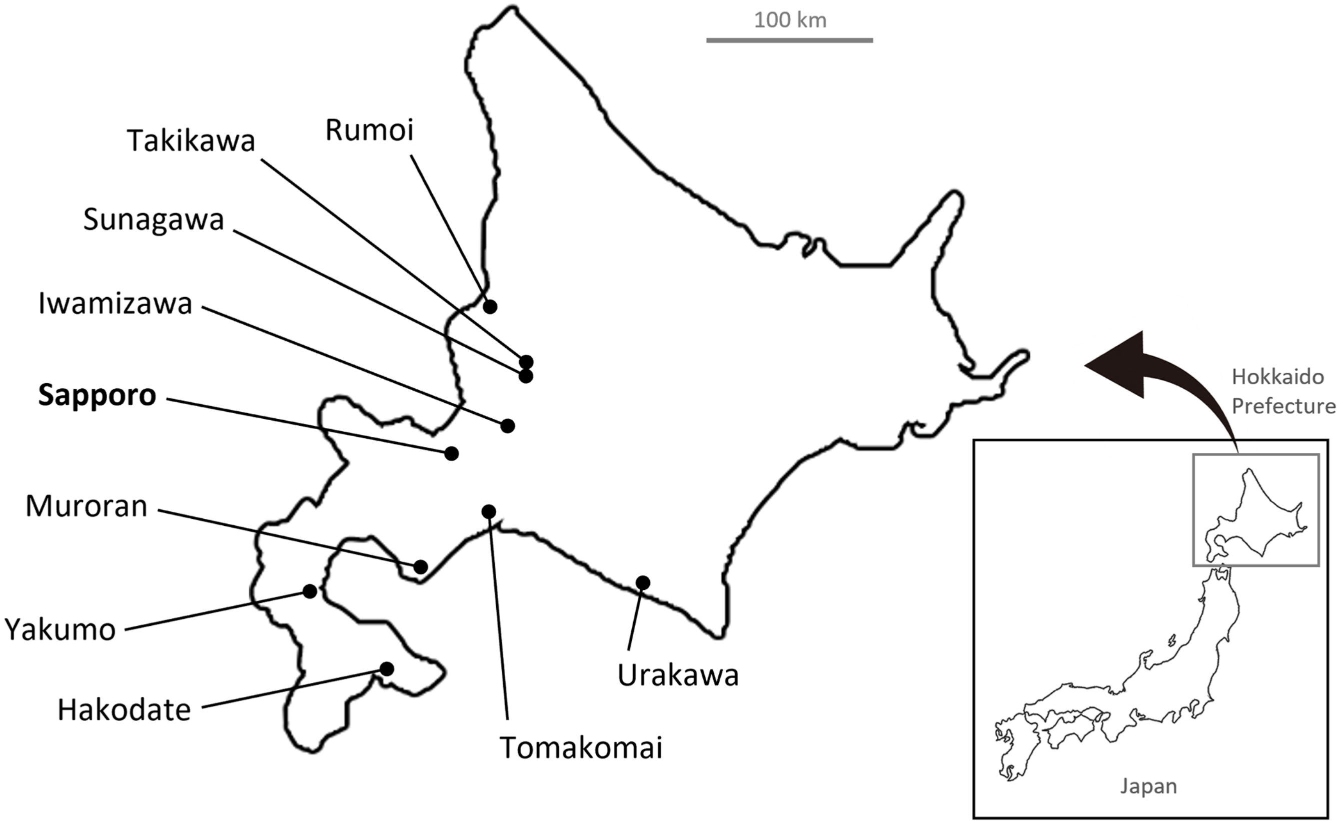

Supplementary Figure S1. A map showing the location of the hospitals where the surveillance was conducted.
