# Supplementary material for: Molecular evolutionary analysis of novel NSP4 mono-reassortant G1P[8]-E2 rotavirus strains that caused a discontinuous epidemic in Japan in 2015 and 2018
Source: Front Microbiol. 2024 Jul 10;15:1430557. doi: 10.3389/fmicb.2024.1430557 (PMC11266183; doi:10.3389/fmicb.2024.1430557)
Supplement: Supplementary Figure S2 — RVA genotype distributions for each hospital (2014–2019). Three hospitals, namely, NTT Medical Center Sapporo (NTT), JCHO Sapporo Hokushin Hospital (JSH), and Hokkaido Medical Center (HMC), are located in Sapporo city. Other hospitals are indicated by city name. [file Image_2.pdf]

2014

Hospital	NTT	JSH	HMC	Takikawa	Iwamizawa	Tomakomai	Muroran	Hakodate	Urakawa	Rumoi	Sunagawa	Yakumo	Total
G1P[8] (Wa)	-	4	-	-	0	4	4	-	1	-	-	-	13
G1P[8]-E2	-	-	-	-	-	-	-	-	-	-	-	-	0
G1P[8] (DS-1)	-	1	-	-	4	3	0	-	0	-	-	-	8
G2P[4] (DS-1)	-	2	-	-	0	3	10	-	0	-	-	-	15
G3P[8] (Wa)	-	0	-	-	0	0	0	-	0	-	-	-	0
G3P[8] (DS-1)	-	0	-	-	0	0	0	-	0	-	-	-	0
G8P[8] (DS-1)	-	1	-	-	0	17	3	-	11	-	-	-	32
G9P[8] (Wa)	-	0	-	-	0	10	1	-	8	-	-	-	19
G9P[8]-E2	-	-	-	-	-	-	-	-	-	-	-	-	0
Total	-	8	-	-	4	37	18	-	20	-	-	-	87

2015

Hospital	NTT	JSH	HMC	Takikawa	Iwamizawa	Tomakomai	Muroran	Hakodate	Urakawa	Rumoi	Sunagawa	Yakumo	Total
G1P[8] (Wa)	3	-	-	-	-	-	-	-	-	-	-	-	3
G1P[8]-E2	-	-	-	-	2	8	1	-	-	-	-	-	11
G1P[8] (DS-1)	6	21	-	5	9	12	12	2	4	-	-	-	71
G2P[4] (DS-1)	-	1	-	-	1	1	-	-	-	-	-	-	3
G3P[8] (Wa)	-	-	-	-	-	-	-	-	-	-	-	-	0
G3P[8] (DS-1)	-	-	-	-	-	-	-	-	-	-	-	-	0
G8P[8] (DS-1)	-	-	-	5	-	-	-	-	-	-	-	-	5
G9P[8] (Wa)	-	2	-	2	1	3	-	8	1	-	-	-	17
G9P[8]-E2	-	-	-	-	-	-	-	-	-	-	-	-	0
Total	9	24	-	12	13	24	13	10	5	-	-	-	110

2016

Hospital	NTT	JSH	HMC	Takikawa	Iwamizawa	Tomakomai	Muroran	Hakodate	Urakawa	Rumoi	Sunagawa	Yakumo	Total
G1P[8] (Wa)	-	-	-	-	-	-	-	-	-	-	-	-	0
G1P[8]-E2	-	-	-	-	-	-	-	-	-	-	-	-	0
G1P[8] (DS-1)	-	-	-	-	-	-	-	-	-	-	-	-	0
G2P[4] (DS-1)	-	2	-	-	-	1	-	3	-	2	-	-	8
G3P[8] (Wa)	-	-	-	-	-	-	-	-	-	-	-	-	0
G3P[8] (DS-1)	1	-	-	1	2	19	-	-	2	-	-	-	25
G8P[8] (DS-1)	1	-	-	1	-	-	-	-	-	-	-	-	2
G9P[8] (Wa)	8	29	-	1	11	10	3	2	11	17	-	-	92
G9P[8]-E2	-	-	-	-	-	-	-	-	-	-	-	-	0
Total	10	31	-	3	13	30	3	5	13	19	-	-	127

2017

Hospital	NTT	JSH	HMC	Takikawa	Iwamizawa	Tomakomai	Muroran	Hakodate	Urakawa	Rumoi	Sunagawa	Yakumo	Total
G1P[8] (Wa)	-	-	-	-	-	1	-	-	-	-	-	-	1
G1P[8]-E2	-	-	-	-	-	-	-	-	-	-	-	-	0
G1P[8] (DS-1)	-	-	-	-	-	-	-	-	-	-	-	-	0
G2P[4] (DS-1)		1	-		-			1		1		5	8
G3P[8] (Wa)	-	-	-	-	-	-	-	-	-	-	-	-	0
G3P[8] (DS-1)	3	-	-	8	-	2	-	-	-	3	23	-	39
G8P[8] (DS-1)	1	-	-	-	-	1	-	-	1	1	2	-	6
G9P[8] (Wa)	-	-	-	-	-	1	3	-	-	1	-	-	5
G9P[8]-E2	-	-	-	-	-	-	-	-	-	-	-	-	0
Total	4	1	-	8	-	5	3	1	1	6	-	5	59

2018

Hospital	NTT	JSH	HMC	Takikawa	Iwamizawa	Tomakomai	Muroran	Hakodate	Urakawa	Rumoi	Sunagawa	Yakumo	Total
G1P[8] (Wa)	-	-	-	-	-	-	-	-	-	-	-	-	0
G1P[8]-E2	5	4	-	-	3	14	2	1	-	-	2	-	31
G1P[8] (DS-1)	-	-	-	-	-	-	-	-	-	-	-	-	0
G2P[4] (DS-1)	1	1	-	-	2	1	1	-	-	8	1	-	15
G3P[8] (Wa)	-	-	-	-	1	-	-	-	-	-	1	-	2
G3P[8] (DS-1)	-	-	-	-	-	1	-	-	-	-	-	-	1
G8P[8] (DS-1)	-	-	-	-	-	-	-	-	-	-	-	-	0
G9P[8] (Wa)	-	-	-	-	-	2	6	-	-	-	-	-	8
G9P[8]-E2	1	-	-	-	-	-	-	1	-	-	-	-	2
Total	7	5	-	-	6	18	9	2	-	8	4	-	59

2019

Hospital	NTT	JSH	HMC	Takikawa	Iwamizawa	Tomakomai	Muroran	Hakodate	Urakawa	Rumoi	Sunagawa	Yakumo	Total
G1P[8] (Wa)	2	2	-	-	-	-	-	-	-	6	-	-	10
G1P[8]-E2	-	-	-	-	-	-	-	-	-	-	-	-	0
G1P[8] (DS-1)	-	-	-	-	-	-	-	-	-	-	-	-	0
G2P[4] (DS-1)	2	-	-	-	-	1	-	-	-	-	-	-	3
G3P[8] (Wa)	-	-	-	-	-	-	-	-	-	-	-	-	0
G3P[8] (DS-1)	-	-	2	-	-	-	-	-	-	-	-	-	2
G8P[8] (DS-1)	2	1	1	-	1	1	-	1	-	1	1	-	9
G9P[8] (Wa)	1	2	-	-	-	1	-	8	-	-	-	-	12
G9P[8]-E2	3	1	3	-	-	-	-	4	-	-	-	-	11
Total	10	6	-	-	1	3	-	13	-	7	-	-	47

Supplementary Figure S2. RVA genotype distributions for each hospital (2014–2019).
