# Supplementary material for: Molecular evolutionary analysis of novel NSP4 mono-reassortant G1P[8]-E2 rotavirus strains that caused a discontinuous epidemic in Japan in 2015 and 2018
Source: Front Microbiol. 2024 Jul 10;15:1430557. doi: 10.3389/fmicb.2024.1430557 (PMC11266183; doi:10.3389/fmicb.2024.1430557)
Supplement: Supplementary Table S1 — Genotype constellations of RVA strains detected in Hokkaido, Japan (2014–19). [file Table_1.docx]

Supplementary Table S1. Genotype constellations of RVA strains detected in Hokkaido, Japan (2014–19).

| Type | Genotype^*^ | | | | | | | | | | |
| --- | --- | --- | --- | --- | --- | --- | --- | --- | --- | --- | --- |
|  | VP7 | VP4 | VP6 | VP1 | VP2 | VP3 | NSP1 | NSP2 | NSP3 | NSP4 | NSP5 |
| G1P[8] (Wa) | G1 | P[8] | I1 | R1 | C1 | M1 | A1 | N1 | T1 | E1 | H1 |
| G1P[8]-E2 | G1 | P[8] | I1 | R1 | C1 | M1 | A1 | N1 | T1 | E2 | H1 |
| G1P[8] (DS-1) | G1 | P[8] | I2 | R2 | C2 | M2 | A2 | N2 | T2 | E2 | H2 |
| G2P[4] (DS-1) | G2 | P[4] | I2 | R2 | C2 | M2 | A2 | N2 | T2 | E2 | H2 |
| G3P[8] (Wa) | G3 | P[8] | I1 | R1 | C1 | M1 | A1 | N1 | T1 | E1 | H1 |
| G3P[8] (DS-1) | G3 | P[8] | I2 | R2 | C2 | M2 | A2 | N2 | T2 | E2 | H2 |
| G8P[8] (DS-1) | G8 | P[8] | I2 | R2 | C2 | M2 | A2 | N2 | T2 | E2 | H2 |
| G9P[8] (Wa) | G9 | P[8] | I1 | R1 | C1 | M1 | A1 | N1 | T1 | E1 | H1 |
| G9P[8]-E2 | G9 | P[8] | I1 | R1 | C1 | M1 | A1 | N1 | T1 | E2 | H1 |

^*^Gray color indicates a DS-1-like genotype.
