# Supplementary material for: Molecular evolutionary analysis of novel NSP4 mono-reassortant G1P[8]-E2 rotavirus strains that caused a discontinuous epidemic in Japan in 2015 and 2018
Source: Front Microbiol. 2024 Jul 10;15:1430557. doi: 10.3389/fmicb.2024.1430557 (PMC11266183; doi:10.3389/fmicb.2024.1430557)
Supplement: Supplementary Table S2 — Evolutionary rate of each gene of rotaviruses. [file Table_2.docx]

Supplementary Table S2. Evolutionary rate of each gene of rotaviruses.

| Gene | Type | Clock rate | | No. of | (detected in | Model |
| --- | --- | --- | --- | --- | --- | --- |
|  |  | Mean | 95% HPD Interval | samples | this study) |  |
| VP7 | G1 | 7.67E-04 | (6.61E-04–8.79E-04) | 311 | 107 | HKY+G |
|  | G2 | 7.34E-04 | (6.15E-04–8.67E-04) | 196 | 29 | HKY+G |
|  | G3 | 8.48E-04 | (6.65E-04–1.04E-03) | 156 | 34 | HKY+G |
|  | G8 | 7.28E-04 | (6.30E-04–8.37E-04) | 115 | 17 | HKY+G |
|  | G9 | 1.01E-03 | (8.43E-04–1.20E-03) | 243 | 70 | HKY+I |
| VP4 | P[4] | 6.86E-04 | (5.98E-04–7.78E-04) | 200 | 29 | HKY+G |
|  | P[8] | 7.09E-04 | (5.67E-04–8.36E-04) | 540 | 228 | TN93+G |
| VP6 | I1 | 7.28E-04 | (6.30E-04–8.37E-04) | 349 | 148 | HKY+G |
|  | I2 | 8.11E-04 | (6.98E-04–9.36E-04) | 327 | 127 | HKY+G |
| VP1 | R1 | 5.48E-04 | (4.81E-04–6.17E-04) | 337 | 147 | TN93+G |
|  | R2 | 6.86E-04 | (6.19E-04–7.59E-04) | 326 | 128 | GTR+G+I |
| VP2 | C1 | 5.95E-04 | (5.22E-04–6.74E-04) | 335 | 146 | TN93+I |
|  | C2 | 7.48E-04 | (6.60E-04–8.45E-04) | 323 | 127 | TN93+G+I |
| VP3 | M1 | 7.22E-04 | (6.43E-04–8.03E-04) | 333 | 143 | TN93+G |
|  | M2 | 8.32E-04 | (7.42E-04–9.33E-04) | 324 | 127 | TN93+G+I |
| NSP1 | A1 | 8.72E-04 | (7.55E-04–9.99E-04) | 337 | 149 | HKY+G+I |
|  | A2 | 8.26E-04 | (7.15E-04–9.53E-04) | 320 | 125 | HKY+G |
| NSP2 | N1 | 8.38E-04 | (6.95E-04–9.81E-04) | 338 | 147 | HKY+G |
|  | N2 | 9.27E-04 | (7.81E-04–1.09E-03) | 318 | 125 | HKY+G |
| NSP3 | T1 | 7.93E-04 | (6.77E-04–9.28E-04) | 335 | 145 | TN93+G |
|  | T2 | 9.55E-04 | (8.00E-04–1.12E-03) | 321 | 127 | TN93+G |
| NSP4 | E1 | 8.23E-04 | (6.84E-04–9.76E-04) | 289 | 100 | TN93+G |
|  | E2 | 1.18E-03 | (9.98E-03–1.37E-03) | 456 | 192 | HKY+G |
| NSP5 | H1 | 8.27E-04 | (6.61E-04–1.00E-03) | 329 | 143 | HKY+G |
|  | H2 | 9.43E-04 | (7.67E-04–1.13E-03) | 316 | 126 | HKY+G |
