# Supplementary material for: Molecular evolutionary analysis of novel NSP4 mono-reassortant G1P[8]-E2 rotavirus strains that caused a discontinuous epidemic in Japan in 2015 and 2018
Source: Front Microbiol. 2024 Jul 10;15:1430557. doi: 10.3389/fmicb.2024.1430557 (PMC11266183; doi:10.3389/fmicb.2024.1430557)
Supplement: Supplementary Table S3 — The Times of Most-Recent Common Ancestor (TMRCA) of NSP4 mono-reassortant strains. [file Table_3.docx]

Supplementary Table S3. The Times of Most-Recent Common Ancestor (TMRCA) of NSP4 mono-reassortant strains.

|  | Gene | TMRCA | (95% HPD Interval) | The most recent strain from GenBank |
| --- | --- | --- | --- | --- |
| G1-E2 strains | | |  |  |
|  | VP7 | 2013.2 | (2012.1–2014.2) | Human-wt/VNM/12013_50/2012/G1P[8] |
|  | VP4 | 2013.7 | (2005.1–2014.7) | Human-wt/VNM/12013_46/2012/G1P[8] |
|  | VP6 | 2013.6 | (2012.6–2014.4) | Human-wt/VNM/12013_50/2012/G1P[8] |
|  | VP1 | 2013.2 | (2008.3–2014.5) | Human-wt/VNM/12013_46/2012/G1P[8] |
|  | VP2 | 2013.7 | (2009.6–2014.5) | Human-wt/VNM/12013_46/2012/G1P[8] |
|  | VP3 | 2013.7 | (2012.9–2014.6) | Human-wt/VNM/12013_50/2012/G1P[8] |
|  | NSP1 | 2013.7 | (2007.8–2014.6) | Human-wt/VNM/12013_51/2012/G1P[8] |
|  | NSP2 | 2013.2 | (2012.2–2014.0) | Human-wt/VNM/12067_88/2012/G1P[8] |
|  | NSP3 | 2013.6 | (2012.7–2014.4) | Human-wt/VNM/12013_50/2012/G1P[8] |
|  | NSP4 | 2013.0 | (2012.8–2014.2) | Human-wt/THA/LS-202/2014/G2P[4] |
|  | NSP5 | 2012.9 | (2012.2–2014.2) | Human-wt/VNM/12013_50/2012/G1P[8] |
|  |  |  |  |  |
| G9-E2 strains | | |  |  |
|  | VP7 | 2015.7 | (2015.4–2016.8) | Japanese G9(Wa) strains (this study) |
|  | VP4 | 2016.3 | (2004.1–2017.2) | Japanese G9(Wa) strains (this study) |
|  | VP6 | 2016.4 | (2015.2–2017.2) | Japanese G9(Wa) strains (this study) |
|  | VP1 | 2016.1 | (2008.1–2017.2) | Japanese G9(Wa) strains (this study) |
|  | VP2 | 2016.3 | (2006.4–2017.1) | Japanese G9(Wa) strains (this study) |
|  | VP3 | 2016.5 | (2015.5–2017.2) | Japanese G9(Wa) strains (this study) |
|  | NSP1 | 2016.1 | (2007.5–2017.3) | Japanese G9(Wa) strains (this study) |
|  | NSP2 | 2015.3 | (2014.6–2016.3) | Japanese G9(Wa) strains (this study) |
|  | NSP3 | 2016.0 | (2014.6–2016.9) | Japanese G9(Wa) strains (this study) |
|  | NSP4 | 2014.4 | (2014.2–2015.5) | Japanese G2 strains (this study) |
|  | NSP5 | 2016.0 | (2015.5–2017.4) | Japanese G9(Wa) strains (this study) |
